# Cellular Targeting of Oligonucleotides by Conjugation with Small Molecules

**DOI:** 10.3390/molecules25245963

**Published:** 2020-12-16

**Authors:** Manuel Hawner, Christian Ducho

**Affiliations:** Department of Pharmacy, Pharmaceutical and Medicinal Chemistry, Saarland University, Campus C2 3, 66 123 Saarbrücken, Germany; manuel.hawner@uni-saarland.de

**Keywords:** oligonucleotides, conjugation, small molecules, cellular targeting

## Abstract

Drug candidates derived from oligonucleotides (ON) are receiving increased attention that is supported by the clinical approval of several ON drugs. Such therapeutic ON are designed to alter the expression levels of specific disease-related proteins, e.g., by displaying antigene, antisense, and RNA interference mechanisms. However, the high polarity of the polyanionic ON and their relatively rapid nuclease-mediated cleavage represent two major pharmacokinetic hurdles for their application in vivo. This has led to a range of non-natural modifications of ON structures that are routinely applied in the design of therapeutic ON. The polyanionic architecture of ON often hampers their penetration of target cells or tissues, and ON usually show no inherent specificity for certain cell types. These limitations can be overcome by conjugation of ON with molecular entities mediating cellular ‘targeting’, i.e., enhanced accumulation at and/or penetration of a specific cell type. In this context, the use of small molecules as targeting units appears particularly attractive and promising. This review provides an overview of advances in the emerging field of cellular targeting of ON via their conjugation with small-molecule targeting structures.

## 1. Introduction

### 1.1. Principle Biological Mechanisms of Oligonucleotide Action

**Figure 1:**
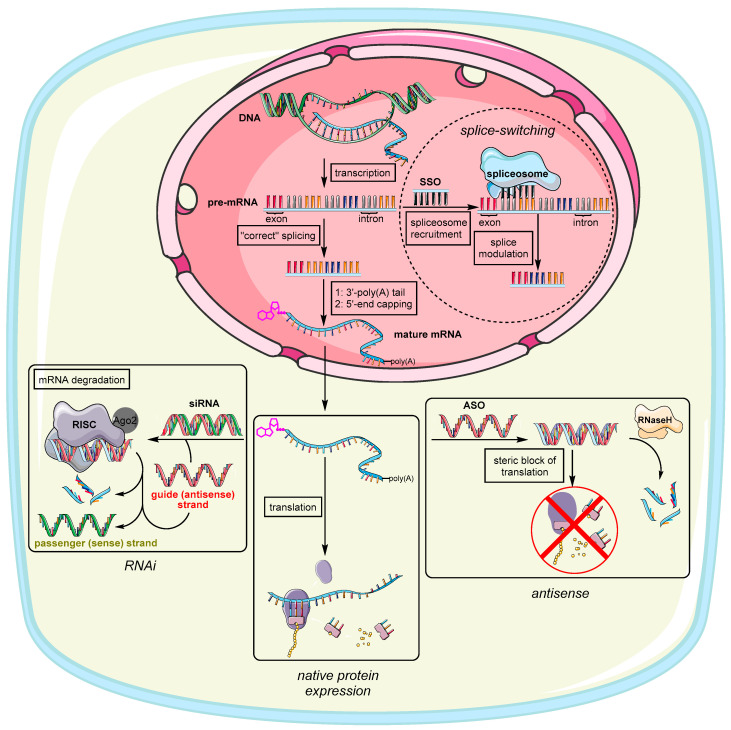
Principle modes of action of therapeutic ON in the nucleus and the cytosol in relation to native (i.e., unaltered) protein expression. This figure uses graphical templates from Servier Medical Art, licensed under a Creative Common Attribution 3.0 Generic License. http://smart.servier.com/.

The earliest steps to address genetic disorders or infections at the genetic level have been made with so-called antisense oligonucleotides (ASO) (Figure 1). The potential to exploit exogenous deoxyribonucleic acids for the purpose of medical treatment were discovered by Zamecnik and Stephenson in association with Rous Sarcoma Virus [1], where single strand (ss) DNA was applied to form a DNA/RNA heteroduplex with a complementary viral RNA via Watson–Crick base pairing [2]. In general, the emergence of any irregular biological activity [3,4] is often related to a homeostatic imbalance of genes [5] or proteins [6] within cells or tissues. Targeting mRNA as the fundamental blueprint and source of pathology therefore represents an attractive option for restoring balance and decommissioning the expression of defective and dilapidated genes or their protein products. In general, a synthetic oligonucleotide (ON) can induce its action in two different cell compartments: the cytoplasm and the nucleus [7]. Interferences with genetic material in the nucleus are summarized under the collective term ‘antigene’ and are beyond the scope of this review [8,9]. However, antisense action takes place in the cytoplasm where ASO compromise translation of mRNA via two common pathways [10]. On the one hand, the tailor-made sequence binds to its ribonucleic counterpart causing simple translational arrest due to steric hindrance at the ribosomes’ binding site or by prohibiting ribosomal motion along the mRNA [11]. On the other hand, the ASO might recruit the endonuclease RNase H that selectively recognizes RNA in DNA/RNA heteroduplexes and degrades the RNA strand [12]. Because of this inherent feature of RNases [13], ASO are only required in small ‘catalytic’ amounts, which helps with several pharmacokinetic obstacles to ON delivery that are described in the following sections. ASO are nowadays often designed as chimeric gapmers, which have deoxyribonucleotides in the center flanked by several modified nucleotides to prevent unwanted degradation by exonucleases [14,15,16]. They often also possess a phosphorothioate backbone further enhancing serum stability as the sulfur-containing modification impedes nuclease activity [17,18]. Phosphorothioate ON exhibit an enhanced binding to serum proteins [19], which is why such ON are preferably taken up by the liver [20,21]. Two known FDA-approved representatives of this type are Fomivirsen [22] (against cytomegalovirus retinitis, the first FDA-approved ASO drug) and Mipomersen [23] (against familial hypercholesterolemia).

Splice-switching ON (SSO) are a variation of ASO that interact with nuclear pre-mRNA during the splicing process, thus altering exon inclusion, exclusion, or choice in general (Figure 1) [24]. SSO are usually administered to treat genetic disorders, which are consequences of genetic mutations within these exons [25]. For this reason, there are many starting points for SSO to literally splice out defective genes. Exons in general are the coding sections of genes interspersed with introns, i.e., non-coding sequences within the pre-mRNA transcript [26]. During the splicing process, the exons containing the transcript for proteins are fused together by the spliceosome, thus leaving out the introns [27]. The spliceosome therefore has to bind to specific splicing sites, by which the order of exon combination can also be altered (alternative splicing) [28]. This offers two options to restore correct splicing. First, an exogenous ASO is designed to base-pair to a (non-natural) splicing enhancer sequence [29] created through mutation [30]. Blocking this site causes the machinery to skip this exon leaving out genetic disinformation. Second, an ASO can be designed to base-pair with a splicing silencer sequence. This prevents binding of a negatively acting splicing protein, ultimately causing inclusion of original exons that were skipped before (through mutation) [31]. Approved SSO therapeutics are Nusinersen [32,33] for the treatment of spinal muscular atrophy, Golodirsen [34,35] and Eteplirsen [36,37] to treat Duchenne muscular dystrophy, and Milasen [38], a pioneering example of personalized medicine to treat Mila Makovec’s form of batten disease.

The discovery of RNA interference (RNAi) as a fundamental endogenous mechanism for controlling gene expression further broadened the potential of therapeutic ON and served as a boost in the development of new ON structures [39]. In RNAi, small non-coding double-stranded (ds) RNA (usually 20–25 nucleotides long) targets mature mRNA in the cytosol to prevent the expression of specific proteins by mediating the degradation of mRNA (Figure 1) [40]. The original physiological functions of RNAi are widespread, e.g., degradation of exogenous viral RNA [41] or regulation of endogenous gene expression. These processes are mediated by endogenous versions of the aforementioned dsRNA usually referred to as microRNA (miRNA) [42]. To achieve miRNA-mediated RNAi, long dsRNA is cleaved by dicer proteins to generate shorter dsRNA fragments consisting of a sense (passenger) and antisense (guide) strand. The latter of these two strands is complementary to the target mRNA sequence that is supposed to be degraded. This step is necessary to prevent interferon-mediated immune response [43]. The guide strand and the target mRNA are recognized by ribonucleoprotein complexes named RNA-induced silencing complexes (RISC) [44]. During the formation of RISC, endonucleases known as Argonaut proteins (Ago2) separate the guide (antisense) strand from its passenger (sense) strand, thus only loading the guide strand into the RISC [45]. Artificial exogenous versions of mature miRNA are usually referred to as small interfering RNA (siRNA). For siRNA to get Ago2 to choose the ‘correct’ strand as guide strand, non-canonical modifications are imbedded in the backbone of the passenger (sense) strand that are not compatible with the RISC machinery [46]. Ultimately, the preferred incorporation of the desired (guide) strand into the RISC can be steered by two factors. First, lowering duplex stability simplifies strand separation by Ago2. Second, pronounced chemical modifications of one strand can hamper its incorporation because it does not meet the structural requirements of the RISC [46,47]. RNA exhibiting an A-form helical topology is generally preferred for entering the RISC [47]. Finally, the targeted mRNA that is complementary to the guide strand is cleaved between nucleotides 10–11 (counted from the 5′-end) [48]. This therefore represents an adequate binding site for modifications and targeting ligands, further enhancing strand separation and altering the silencing of protein expression [49,50]. This is demonstrated in several examples [51,52,53,54,55,56]. A prominent representative of RNAi-triggering therapeutics is the first FDA-approved siRNA drug Patisiran [57,58] against hereditary transthyretin-mediated amyloidosis. Another promising candidate in a phase III clinical trial is Inclisiran [59,60] (for the treatment of familial hypercholesterolemia). Very recently, the FDA [61] as well as the European Medicines Agency [62] have approved Lumasiran as a novel RNAi drug that will be used for the treatment of primary hyperoxaluria type 1 (PH1). This ‘orphan status’ conjugate consists of an siRNA that targets hydroxy acid oxidase 1 (HAO1) mRNA and that is covalently bound to a ligand containing three *N*-acetylgalactosamine (GalNAc) units for improved delivery [63]. The presence of a defective variant of HAO1 leads to nephrocalcinosis and calcium oxalate crystal deposition in the urinary tract, which causes kidney damage and urinary tract infections and, in the worst case, may thus require an organ transplant [64,65]. More siRNA drug candidates that are currently in clinical trials have been reviewed by Song et al. [66].

### 1.2. Chemically Modified ON

Despite the tremendous potential that ASO, SSO, siRNA and other ON-based therapeutics [7,67] exhibit in vitro, they are subject to a range of hurdles in vivo. These are mainly related to distribution, metabolism and toxicity [68]. For instance, synthetic oligonucleotides are potential substrates for exo- and endonucleases, leading to short half-lives in vivo [69]. Their native polyanionic structure results in poor uptake into target cells or tissues [70]. Extracellular degradation in blood and tissue, intracellular degradation in the reticuloendothelial system (RES) as well as loss of ON by rapid renal excretion further limit their therapeutic potency in vivo and can ultimately lead to their ineffectiveness [71]. To overcome these hurdles, a plethora of structural modifications have been established and are summarized below.

**Figure 2:**
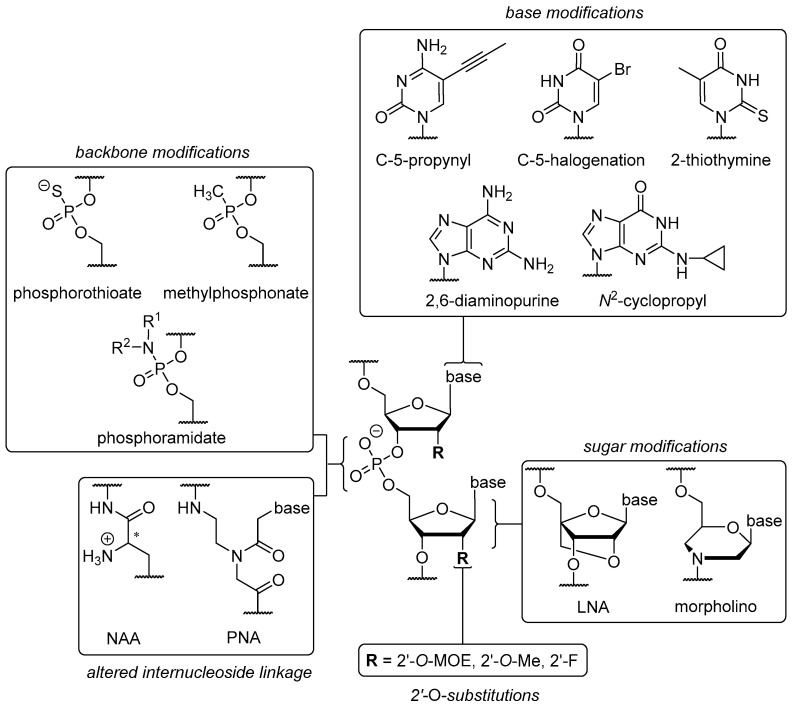
Some selected structural modifications of therapeutic ON with respect to their site of implementation.

The chemical ‘toolbox’ available for ON modifications (Figure 2) can be divided into four sections: (i) modifications of nucleobases; (ii) modifications of internucleoside linkages; (iii) modifications or replacements of the ribose unit; and (iv) alternative backbone structures. Generally speaking, each modification has an underlying rationale and causes improvement to at least one of the aforementioned issues. Depending on their difference to native ON structures and time of publication, several modifications are also grouped in ‘generations’ ranging from first to third.

A modification that is indispensable and has been employed in the structures of Fomivirsen and Mipomersen is the phosphorothioate (PS) backbone (Figure 2). The introduction of non-bridging sulfur at the phosphate unit furnishes a novel stereogenic center at the phosphorus atom [72], resulting in diastereomers with either (*S*_P_) or (*R*_P_) configurations. These show different properties in terms of nuclease stability and binding affinity to the complementary (target) strand [17,18]. The ‘stickiness’ of sulfur further enables binding to plasma proteins such as albumin [73] or other biopolymers like heparin [74]. As a result, it leads to prolonged plasma half-lives [75] and reduced renal excretion. For PS backbones in ON, one has to consider possible toxic side effects though [76]. Together with the electroneutral methylphosphonate modification [77], the PS backbone belongs to the group of so-called ‘first generation’ modifications. Increased nuclease stability can also be achieved via electronic properties contrary to native ON structures, for instance by introducing positively charged nucleoside analogues [8,78] to create partially zwitterionic backbone structures [79,80,81]. A site-specific introduction of such cationic nucleosyl amino acid (NAA)-modifications enhances the stability of the resultant partially zwitterionic ON towards 3′- and 5′-exonucleases and in more complex biological media (human plasma, human cell lysates).

‘Second generation’ modifications are briefly discussed below. However, quite extraordinary backbone structures can be found in the ‘third generation’, e.g., phosphorodiamidate morpholinos (PMO) [77] and peptide nucleic acids (PNA, Figure 2) [82,83]. PMO have a six-membered morpholine ring replacing the ribose unit, thus leading to an N3′→P5′ internucleoside linkage and an electroneutral P-N bond (hence a diamidate). The latter carries lipophilic alkyl chains for enhanced membrane permeability and replaces the anionic oxygen of the native phosphate diester. Due to their significant structural differences to native ON, PMO are not able to trigger RNA degradation via RNase H or RNAi [84]. However, they are successfully used to block RNA interactions or further RNA processing in many ways [85], e.g., splicing of pre-mRNA in the nucleus [86]. The unnatural structure of PMO also causes high nucleolytic stability [87] and prevents toxic side effects correlated with administration [88]. In PMO and PNA backbones, both the ribose unit and the internucleoside linkage are altered. In contrast, locked nucleic acids (LNA, third generation) are considered as sole ribose modification and are characterized by the rigid 3′-*endo* (North, N) conformation in which a methylene group bridges the 2′-O-substituent and the C-4′ position [89], hence leading to a *gauche* conformation [90]. This rigidified sugar structure has been proven to enhance duplex stability [91] when hybridized with other LNA, DNA, and RNA strands [92,93]. Furthermore, LNA shows enhanced stability against exonuclease degradation when introduced at the 5′-end [94] or the 3′-end [15], thus displaying a key property for successful drug delivery. LNA are often used in so-called ‘gapmer’ structures. In gapmer ON, LNA-modified ends flank a less modified (e.g., PS) central ‘gap’ segment, hence furnishing enhanced nuclease stability with retention of RNase H activation [14].

Some notable ‘second generation’ modifications are placed in the 2′-position of the ribose ring, e.g., (i) *O-*alkylations such as 2′-*O*-methyl (2’-OMe), a naturally occurring RNA sugar modification [95] and (ii) artificial 2′-*O*-methoxyethyl (2’-MOE) moieties (Figure 2) [96]. Both of these modifications are commonly implemented in the design of ASO gapmers. Due to stereoelectronic effects, they are not suitable to promote RNase H activation [97]. However, the same properties as well as the electron-withdrawing oxygen atom also contribute to an N-type/*gauche* conformation [98]. Hence, the formation of an A-helix is promoted [99] that shows increased nuclease stability [100] due to elevated melting temperatures [101] and bound water molecules in the minor groove (2′-MOE) [98]. Placing a fluorine atom at the 2′-position affords unique properties for RNAi. 2′-Fluoro-RNA does not cause immunostimulatory effects [102]. As a result of the polarized C-F bond and the absence of H-bond interactions in the minor groove, the 2′-fluoro modification leads to an augmented enthalpy [103] causing stronger Watson-Crick base-pairing [104] that ultimately results in more stable duplex formation [105]. It also furnishes significantly improved nuclease stability [106] resulting from a preferred 3′-*endo* (North) conformation.

The overall goal of structural changes to the nucleobases usually is an improvement of both pharmacodynamics and pharmacokinetics, i.e., enhanced duplex stability and target specificity, nuclease stability, and improved cellular uptake. These goals have to be met with retained Watson-Crick base pairing fidelity. However, impact of the modified bases depends both on the choice of its placement in the guide or passenger strand of siRNA [107] and within a strand (i.e., modifications at the 5′-end or 3′-end) [108]. Thus, improvements in one property can be linked to a deterioration in another one. Possibilities for structural changes to the nucleobases are numerous as summarized, for instance, by Herdewijn et al. [109] and Peacock et al. [110]. Hence, only a few selected examples are briefly mentioned here [111,112,113,114].

In the case of pyrimidine bases, structural alterations of the scaffold can be made at four positions, i.e., at C-2, C-4, C-5, and C-6. Variations at the C-5-position represent a favorable option for improved RNAi activity and duplex stability. Such C-5-variations can be, for example, halogenated derivatives such as 5-bromouracil (U[5Br]) and 5-iodouracil (U[5I], Figure 2) that are capable of tautomerism [115] due to their acidified *N*^3^-hydrogen. The original aim was to strengthen H-bonding and improve base stacking. Unfortunately, different base-pairing properties were found to be possible depending on whether the keto or enol tautomer is present. This, in turn, depended on the pH of the environment [116]. The keto tautomer is able to base-pair with adenine (Watson-Crick type), stabilizing A-U H-bonding relative to unmodified U, giving decreased RNAi activity [115]. The enol tautomer is able to mimic cytosine and thus pairs with guanine. This leads to point mutations and unfavorable mismatches [117]. Replacing the methyl group in thymine did not show any advantage [118], so C-5 halogenations seem to have no decisive benefit. C-5-alkyl cytosine analogues, in particular naturally occurring C-5-methyl derivatives, proved to increase melting temperatures by 0.5 °C per modification [119]. This feature originates from hydrophobic methyl stacking between the planar nucleobases in the major groove. The modification also reduces immunostimulation and retains nuclease stability [111]. C-5-methyl derivatives of uracil and cytosine lead to retained or improved silencing activities, depending on their site of implementation [120]. Unfortunately, they are prone to deamination in acidic environment [121]. The incorporation of 5-ethyl cytosine led to less stable duplexes with reduced affinity compared to 5-methyl and native cytosine [122] as well as to a reversible and fast transition between B- and Z-helices. However, C-5-propynyl derivatives of uracil and cytosine afforded enhanced cell permeability and duplex formation in ASO [123] due to the alkyne unit stacking in the major groove. On the other hand, this modification reduced RNAi activity when placed at the guide strand’s 5′-end [120]. Contrary results alongside good base-pairing fidelity were achieved for placement of the modification within this strand [124]. Improved duplex stability and RNase H activation were shown for C-5-heteroaryl substituents such as thiazole, pyridine, thiophene, and imidazole. These showed coplanar orientation with the pyrimidine moiety and thus base stacking with neighboring adenine [125]. Unaffected H-bonding in the major groove is a general requirement for effective RNAi [115,120]. The introduction of 2-thiothymine within the gap region caused RNase H-mediated cleavage at an altered position within the mRNA strand. This correlated with a steric clash with some amino acids in the active site [126]. Furthermore, the highly polarizable sulfur atom stabilized the 3′-*endo* conformation [127] leading to stronger H-bonding [128] due to an increased acidity of the 3-NH [129].

In the case of purine bases, the main alterations are introduced at nitrogen atoms in positions 1, 3, and 7, respectively, in addition to C-2, C-6, and C-8. For example, 2,6-diaminopurine (DAP), an adenine analogue with an additional amino group at C-2 (Figure 2), exhibits stronger base-pairing with T due to the formation of three (instead of two) H-bonds, thus overcoming the relative weakness of A-T base-pairing [130,131]. Melting temperatures were increased by 1.5 °C per modification when DAP base-paired with both DNA or RNA [132]. For the incorporation of DAP in ASO, one has to consider that it is prone to depurination under acidic conditions during DMTr-cleavage in ON synthesis. An additional introduction of an *N*^2^-imidazolylpropyl moiety in DAP strongly enhanced duplex stability without impeding RNase H activation at several positions within an ASO strand due to interactions with the phosphate backbone in the minor groove [133]. Replacing the N-7 in adenine by a propynyl group led to increased stability at the expense of reduced activity. An according guanine analogue showed enhancement considering both features [134]. Reduced immunostimulation was found for *N*^2^-cyclopropyl and *N*_2_-propyl modifications, along with retained silencing effects [135]. For this, several positions for an optimal placement in the guide and passenger strands of siRNA were evaluated. *N*^2^-imidazolylpropyl [133] and *N*^2^-aminopropyl derivatives of guanine [136] afforded strongly improved thermal stabilities and retained RNase H activation when placed in DNA or RNA, due to interactions with the phosphate backbone of the counterstrand.

### 1.3. Endocytotic Pathways

Cells have several pathways for the internalization of large ‘biologics’, e.g., clathrin-coated pits, caveolar pathways (lipid rafts with high contents of sphingomyelins, glycosphingolipids, and cholesterol [137]), pinocytosis and phagocytosis [138,139]. However, the interim destination of the thus transported molecules is the same for all endocytic mechanisms: early endosomes (EE). EE are slightly acidic [140], spherical lipid vesicles in which compounds are loaded and sorted [141] for the purpose of transport [142]. Thus, EE can be interpreted as ‘shuttles’ needed for ON to enter cells. The route that transports materials to the trans Golgi network (TGN) is also operated by EE [143]. Once loaded, vesicles from the plasma membrane containing both the ON and an uptake-mediating receptor are translocated to EE. From EE, transport continues towards late endosomes (LE) [144] into which the cargo is reloaded via invading intraluminal vesicles (ILV) [145]. At this stage, LE containing several ILV are commonly referred to as multivesicular bodies (MVB) [144]. Furthermore, EE are also able to mature directly to LE [142] pointing out that EE can transfer cargo via a dual mechanism [146]. Beyond that, Clathrin-dependent cargos have the ability to recycle back to the cell surface via recycling endosomes (RE) [147]. Candidates for immediate return to the plasma membrane are LDL receptors [148], transferrin [149], and the asialoglycoprotein receptor (ASGP-R) [150]. LE ultimately fuse with lysosomes (LY) [151], the primary digestion apparatus of the cell containing degrading enzymes at low pH. Here, ON are ultimately degraded if they have not ‘escaped’. Every point of transfer along this line therefore represents an opportunity for ON to escape to the cytoplasm, where they can unleash their biological activity [152]. In general, the biological action of ASO, SSO, and siRNA correlates with the endocytotic pathway by which they are taken up [153]. siRNA are usually transfected with carriers, e.g., cationic polymers (PP) or polymer nanoparticles (LNP). ASO and SSO, respectively, benefit from their (often employed) PS backbone. However, the release of ON from MVB and/or LE appears to be the crucial endogenous process for attaining pharmacological effects [139].

Regarding the distrubution and cellular uptake of native ON, insufficient delivery is an intrinsic issue due to the lack of structural units that could mediate interactions with cell surfaces or plasma proteins. However, PS-modified ON show a boost in cell penetration via clathrin-dependent pathways as they directly bind to cell surface receptors (stabilin [21], scavenger receptors [154]) or interact with a vast number of proteins [155] such as annexin A2, thus additionally enhancing ASO activity. Once taken up, they can be transported from EE to LE and LY [156]. The release of such therapeutic ON usually occurs from LE [157] via back fusion processes in which lysobisphosphatidic acid (LBPA)-rich ILV are suggested to be bound to the LE membranes [158,159], as it is discussed in more detail below.

Apart from non-conjugated therapeutic ON, whose cellular uptake is commonly referred to as ‘gymnosis’, ON therapeutics are often administered with additives. For siRNA, most of the early approaches in this direction were based on the formation of large aggregates, e.g., lipid and synthetic nanoparticles, rather than on structurally modified siRNA with defined molecular composition. For instance, siRNA embedded in neutral LNP has been shown to mainly enter the endosome via macropinocytosis and break free from a hybrid EE/LE before being translocated to LE or LY [160]. siRNA wrapped in cationic LNP also uses micropinocytosis for cell entry and is then delivered to LE and LY [161]. A crucial finding was the key role of the recycling machinery for all sorts of endosomes regarding intracellular delivery. Herein, the cholesterol transport protein NPC1 proved to be involved in the return transport of the internalized ON from LE to the plasma membrane, ultimately lowering the intracellular availability of ON for sufficient biological effects [162]. The uptake of polyplexes of siRNA and cell-penetrating peptides (CPP) as well as of siRNA-CPP conjugates was found to be dependent on scavenger receptors (SCARA) being involved in receptor-mediated endocytosis [163]. Similar observations were made with an SSO, for which clathrin-mediated uptake was responsible for internalization of SSO-CPP polyplexes [164]. Differences were found for the release modalities of an ASO targeting human tyrosine-protein kinase (src) [165] when transfected either as a lipoplex or as a polyplex [166]. The formulation as lipoplex showed a more gradual release from endosomes due to a proposed pore-forming mechanism rather than a fusion process with the endosomal membrane [167]. The polyplex formulation caused an instantaneous release (‘burst’) after internalization owing to a proton-sponge effect, indicating that LE with their inherently lower pH might be the likely sites of escape [140,168]. Spherical nucleic acids (SNA) offer another design opportunity for the delivery of siRNA or ASO via their attachment to nanoscale metal particles, e.g., gold or quantum dots (QD) such as CdSe/ZnS 630 [169]. Studies in C166 mouse endothelial cells revealed caveolar uptake [170]. Final accumulation of such particles in LE was observed, and degradation took place with release of the ON fragments to the cytosol and retention of the core structures inside the endosomes [171]. Recent work showed that the uptake of SNA via clathrin-mediated endocytosis is possible [172] when the class A macrophage scavenger receptor (SR-A) is addressed [173].

Overall, all the aforementioned approaches have in common though that they require vast amounts of additives, thus lowering the drug-load capacity and increasing the risk of adverse side effects. Therefore, the modulation of endocytic pathways towards a uniform uptake mechanism (e.g., receptor-mediated uptake) could improve the biological activity of all types of ON-based therapeutics by turning off the plethora of different uptake mechanisms that (at least partially) contribute to the intracellular endosomal arrest of ON. The conjugation of ON with suitable small molecules can be a way to address cell-specific surface receptors that are mainly internalized via clathrin-mediated processes [174,175], thus enabling a unified uptake pathway (‘self-delivery’) without the use of potentially toxic transfecting agents. For instance, SSO conjugated with an RGD peptide induced receptor-mediated uptake via interaction with integrins, were transported to LE and exhibited a slow, gymnosis-like intracellular release pattern [176]. An siRNA-cholesterol conjugate was found to be internalized via binding to epidermal growth factor at the membrane and to be transported via a specific form of EE having the early endosome antigen 1 (EEA1) [177], a protein required for fusion processes with LE [178].

With respect to endosomal release, it is known that ASO accumulate in late endosomes and that the release of a tiny amount of them [179] enables the downregulation of target mRNA [159,180,181]. However, any form of exogeneous ON have to encounter the complex maze of intracellular pathways as outlined above, thus leading to a variety of intracellular destinations. Upon receptor binding, the ligand-receptor complex begins to constrict in a vesicle, being squeezed out of the plasma membrane with the help of the GTPase dynamin [174] that tightens its spiral structure around the neck of the budding vesicle until the membrane is torn [182]. Initial uptake is then followed by traffic to a number of low pH endomembrane compartments as mentioned above, namely EE, RE, LE, LY, and MVB, but also the TGN and the endoplasmic reticulum (ER) [183]. This trafficking is regulated by other GTPases, the Ras-related in brain (Rab) protein family that is also responsible for cargo selection, promotion of vesicle movement and initiation of fusion processes with target compartments [183,184]. Herein, Rab5 is associated with the movement of clathrin-coated vesicles to the early endosome, Rab9 with traffic from LE to the Golgi apparatus. Subsequently, Rab proteins interact with tethering proteins [185] that mediate recognition of the new vesicles and direct them to the intended destination, e.g., specific Golgi sub-compartments [186], where binding of vesicle membrane-bound v-SNARE (soluble *N*-ethylmaleimide-sensitive factor attachment receptor) to target membrane-bound t-SNARE finally initiates the membrane fusion of cell compartments [187,188]. At this point, inhibition of protein kinase A with several small molecules [189] can prevent trafficking to LY as reaching the Golgi apparatus instead is a key interim step for endosomal escape of ON by intervention of the mannose-6-phosphate receptor (M6PR) [190]. With respect to potential sites for endosomal escape, three routes of vesicle traffic appear to be promising: (i) from RE to the plasma membrane; (ii) from EE to the Golgi apparatus, also known as the retrograde pathway [191]; and (iii) during formation of ILV within MVB, because the hot spots for fusion processes and vesicle budding are situated there and lipid bilayer disruptions are likely to occur at this stage [192]. In case of the latter, LBPA and specific multiprotein complexes (endosomal sorting complexes required for transport, ESCRT) appear to play important roles in the release of intraluminal ON to the cytosol [193]. Herein, ESCRT III promotes the invagination of a membrane bud in the endosome [194] and carries out vesicle biogenesis for entering as well as mediating back fusion [195] together with LBPA [196]. At this point, intermediate non-bilayers are formed, ultimately leading to ON leakage due to strong membrane bending [197] and inner membrane strain of the small ILV [198].

Taken together, the aforementioned findings strongly support efforts to optimize the efficacy of AON or siRNA therapeutics via modulation of their pathways for cellular uptake and intracellular trafficking. The conjugation of ON with cell-targeting small molecules is an innovative approach to achieve at least some of these ultimate goals in the field of therapeutic ON. This review is intended to explore this promising strategy in more detail and to provide an overview of its state-of-the-art.

## 2. Cellular Uptake of ON-Small Molecule Conjugates via Receptor Interactions

Some of the major hurdles for the development of highly potent ON therapeutics are poor cellular uptake and the absence of cell specificity. Furthermore, potentially toxic transfecting agents also are a considerable issue of current strategies for ON delivery. Receptor-mediated endocytosis triggered by small molecule (SM) targeting units (i.e., receptor ligands) offers a promising way to overcome these limitations. For the design of such systems, suitable ligand-receptor pairs have to be evaluated with respect to (i) high affinity of the ligand for the specific receptor; (ii) differences in the expression pattern of the receptor in targeted cell types; (iii) receptor abundance; and (iv) receptor cycling between the plasma membrane and endosomal compartments. This principle understanding of receptor biochemistry is essential for the construction of a potent ON-SM conjugate.

### 2.1. Anisamide Conjugates

Anisamide is a high-affinity ligand of sigma-receptors (σ_1_ and σ_2_) that are found at the endoplasmic reticulum and as transmembrane proteins [199]. These are well-known membrane proteins with neuro-modulatory and ion-channeling roles [200]. They show high affinity for psychostimulants [201] such as cocaine and methamphetamine (σ_1_) or benzamides such as anisamide [202]. Due to overexpression of these receptors in several types of cancer [203], e.g., breast [204], malignant melanoma [205], prostate [206], and lung [207,208], an exploitation of this ligand-receptor system for therapeutic purposes appears to be a promising strategy. Besides some according σ_1_-ligands that are used as radioimaging agents in diagnostics [202], there are only few examples of anisamide being used for sigma-receptor targeting within the context of delivering therapeutic ON conjugates. In pioneering work, Huang et al. [206] improved the delivery of the widely used chemotherapeutic agent doxorubicin (DOX) using liposomes composed of a phospholipid and cholesterol that contained phosphatidylethanolamine-anchored anisamide. This formulation led to significant tumor growth inhibition in DU-145 cells (prostate) due to inhibition of topoisomerase II by encapsulated DOX as shown by competitive inhibition of sigma-receptors with haloperidol. However, patients would need to be treated with a significant amount of delivery-mediating agents causing toxicological issues [199,209] besides the poor drug-loading capacity [210,211]. Further work by Huang et al. [208] reports the delivery of ASO and siRNA using LPD (liposome-polycationic peptide-DNA) particles containing phosphatidylethanolamine-anchored anisamide into NCL-H1299 cells (lung). The transfected ON or siRNA, respectively, furnished mRNA downregulation of survivin, a member of the IAP-protein family inhibiting apoptosis [212]. This ON-mediated knockout of survivin prompted dysregulation of mitotic spindle checkpoints as well as defects in microtubule assembly and function that ultimately led to mitotic catastrophe of the studied cancer cells [213]. However, a set of transfecting agents had to be used here again to achieve the desired effect.

In contrast to these approaches, simple direct conjugation of anisamide to the therapeutic ON leaving out delivery agents can accomplish this goal as well and afford even better results without the aforementioned toxicological concerns. Juliano et al. constructed mono- and multivalent conjugates of anisamide attached to the 5′-end of a 3′-TAMRA-labelled 20-mer PS ASO (2′-*O*-methyl-RNA) [214] targeting a luciferase reporter gene by splice-switching [215]. The anisamide moiety was directly attached to the ON on resin by conventional solid phase-supported chemistry using a phosphoramidite reagent [216]. At 50 nM, the trivalent conjugate showed a more increased uptake in PC3 cells (2-fold) than the monovalent conjugate (1.5-fold) as compared to the non-conjugated PS ASO without any noticeable toxicity. The induction of splice correction turning on luciferase expression was found to be 4-fold increased using the trivalent anisamide conjugate, strongly indicating a preference for multivalency (the monovalent anisamide conjugate did not show significantly enhanced splice-switching relative to the non-conjugated ASO reference). With respect to the concomitantly improved uptake (2-fold increase) and biological efficacy (4-fold increase) of the trivalent conjugate, there appears to be a clear preference for receptor-mediated endocytosis over other forms of internalization. However, more recent results question the delivery of such conjugates via sigma-receptors due to emerging evidences that sigma-receptors might not be present on cell surfaces [202,217].

### 2.2. Anandamide Conjugates

Anandamide (*N*-arachidonoylethanolamine, AEA) is an endogenous ligand of the vanilloid TRPV1 receptor [218] as well as of the cannabinoid receptor [219] that is particularly expressed in human B-cells such as BJAB [220] and neuronal cells such as RBL-2H3 [221]. These cells are usually difficult to transfect and are therefore attractive for a cell-targeting approach exploiting the aforementioned ligand-receptor interaction with corresponding ON conjugates.

Carell et al. employed a postsynthetic (solution-phase) approach for the attachment of anandamide at the 3′-end of a 20-mer siRNA’s sense strand by the Cu-catalyzed alkyne-azide click reaction (CuAAC). The resultant conjugate was envisioned to target Renilla luciferase in a reporter gene assay [222]. The CuAAC step was performed using azido-triethyleneglycol-substituted anandamide and a C-5-octadiyne 2′-deoxyuridine moiety (reaction not shown). Confocal microscopic imaging of RBL-2H3 cells, appropriate for simulating neurons [223,224], proved efficient uptake of AEA-modified ds DNA as well as AEA-modified siRNA, whereas the non-conjugated references failed. Biological activity was evaluated via the reduced expression of luciferase in comparison with established ON-cholesterol conjugates. This proved the AEA conjugate to be slightly more potent (50% reduction) than the cholesterol conjugate (45% reduction) at 500 nM in RBL-2H3 cells. To further evaluate the potential of this new conjugate towards actual endogenous targets, an assay was set up to determine the reduction of spleen tyrosine kinase (SYK) expression, a target for the treatment of inflammatory disorders [225,226]. AEA was connected to the siRNA required for silencing SYK expression [227] and administered to RBL-2H3 cells, showing the AEA conjugate again to be more active (60% reduction of SYK activity) than the cholesterol conjugate (40% reduction). To further highlight anandamide as a highly promising candidate for cellular targeting, silencing of Renilla luciferase was evaluated in absence and presence of jetPRIME™ as transfecting agent. In a concentration range from 10 nM to 1 μM, the sole AEA conjugate showed higher silencing of luciferase expression, de facto rendering jetPRIME™ useless alongside strongly reduced toxicity in BJAB cells.

**Figure 3:**
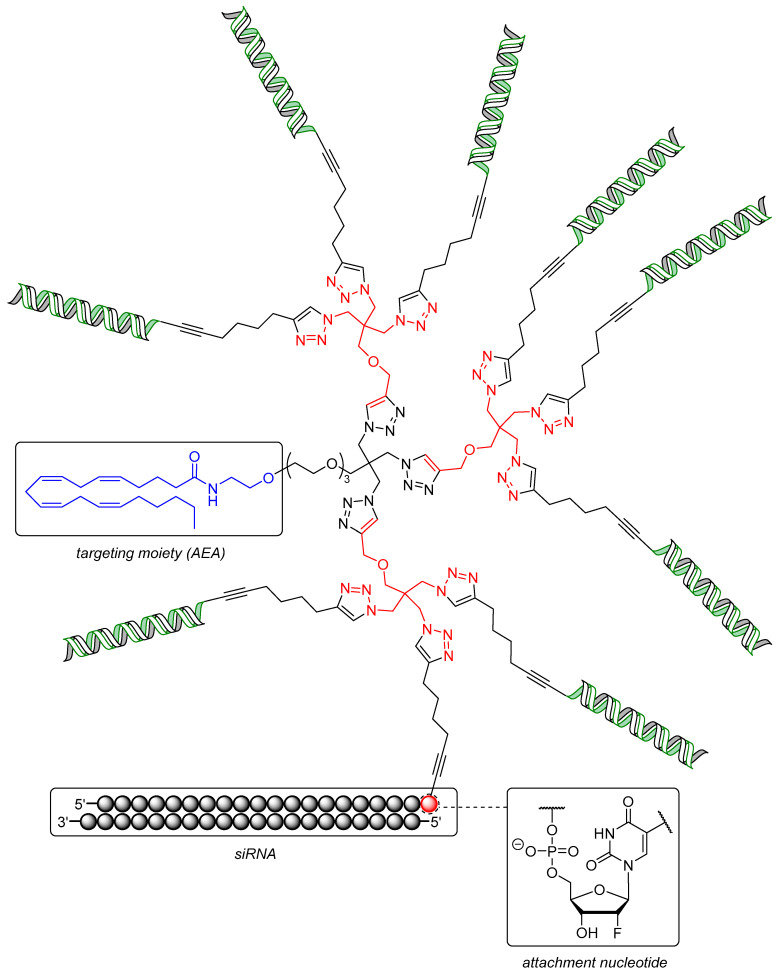
Structure of the dendritic siRNA-containing construct AEA-[9siRNA] equipped with an anandamide (AEA) targeting moiety (blue) and branching units (red: triazido pentaerythritol motif). The siRNA passenger strand (green) is covalently attached to the dendritic scaffold via a uridine-derived attachment nucleotide.

Further improvements of this system towards a set of dendritic siRNA nanostructures targeting the mRNA of nucleo- and phosphoprotein of rabies lyssavirus (RABV) were made by Carell et al. as well [228]. Both proteins are essential for viral replication [229,230] and were targeted by a 20-mer siRNA with AEA attached to it in the same manner as in the aforementioned example (Figure 3) [222]. The crucial building block of the dendrimeric structure was a triazido derivative of pentaerythritol, with three siRNA units coupled to it and one connected AEA moiety to give a trivalent ON construct (AEA-[3siRNA], structure not shown). Three units of a diazido building block coupled to the pentaerythritol analogue led to a final structure with six siRNA units and one AEA moiety (AEA-[6siRNA], structure not shown). Finally, three units of the pentaerythritol derivative clicked to the triazido core yielded a construct with nine siRNA units and one AEA moiety (AEA-[9siRNA], Figure 3). The efficacies of these remarkable structures were first evaluated again using a dual luciferase reporter gene assay in RBL-2H3 cells. Thus, AEA-[3siRNA] showed a decrease of luciferase expression of 50% at one third (166 nM) of the concentration used for monovalent AEA-[1siRNA] (20% at 500 nM), maintaining the overall amount of siRNA equal for the purpose of direct comparison. AEA-[9siRNA] still showed 20% decrease of luciferase expression in spite of a very low concentration of 55 nM. The activity was further improved by attaching one glucose unit to the 3′-end of the antisense strands of AEA-[3siRNA], thus enhancing endosomal stability [231] and affording higher intracellular amounts of ON [232] (AEA-[3siRNA]-Glc, showing 80% decrease of luciferase expression). The activity of AEA-[3siRNA] targeting Tet1 (that is involved in differentiation processes in ENC1 neuronal stem cells [233]) was again found to be higher than the monovalent conjugate, displaying 50% inhibition at 125 nM while unmodified siRNA failed.

Finally, RABV-infected E14 cortical neurons, which are usually difficult to transfect [234,235], were tested with AEA-[3siRNA]-Glc targeting viral nucleo- (N) and phosphoproteins (P). The titers of both N- and P-proteins of RABV were reduced by two orders of magnitude relative to scrambled AEA-siRNA and by one order of magnitude compared to siRNA transfected with Lipofectamine™ 2000 at 1 μM. Overall, monodisperse conjugates of small molecules covalently attached to siRNA dendrimers potentially enable efficient gene silencing in cells that are difficult to address otherwise.

### 2.3. Folate Conjugates

Folate (folic acid, FolA) is an important member of the vitamin B family and represents an essential nutrient participating in the biosynthesis of purines and pyrimidines [236] as well as in amino acid metabolism as a supplier of C-1 building blocks [237]. Several tissues express miscellaneous folate transport proteins such as PCFT [238], RFC [239], and three isoforms of folate receptors (FR) [240]. Among the latter, isoform α is the most abundant congener regarding cancer [241]. In general, FR are cell surface glycosyl phosphatidylinositol (GPI)-anchored glycoproteins to which folate has high affinity (K_D_ = 0.1 nM) [242]. FR are overexpressed in different cancers, e.g., ovaries [243], oral carcinoma [244], malignant pleural mesothelioma [245], and breast cancer [246], owing to the need of growing tumors for high amounts of folate to enable rapid cell division. This makes FR promising entities for the cellular targeting of cancerous tissues. Consequently, conjugates derived from folic acid are already extensively used in drug delivery systems against cancer [247,248] and proved adequate for directed drug delivery. In this context, major challenges are uptake selectivity in desired tissues, thus avoiding unspecific distribution to non-malignant cells, and the potential toxicity of transfecting agents if antitumor ON therapeutics are envisioned to be used. Targeting FR might enable to overcome both of these hurdles because folate-conjugated therapeutics enter cells only via interaction with FR [249,250], and these are considerably overexpressed in many types of cancer. Non-malignant mammalian cells predominantly use transport proteins such as RFC for folate uptake, and these particularly bind reduced forms of the folate ligand [251]. In general, folate is an abundant and well-tolerated effector that is efficiently distributed in humans.

In a proof-of-concept study, Dohmen et al. [244] constructed a monodisperse FolA-PEG ligand that was coupled (by CuAAC) to a 22-mer siRNA having a bio-cleavable alkyne moiety at the 5′-end of its sense strand. The siRNA was designed to silence a reporter GFP-luciferase fusion gene in FR-positive KB/GFPLuc cells [252,253,254] to demonstrate efficacy of the conjugate. Internalization of the FolA-PEG-siRNA conjugate was confirmed by fluorescence microscopy with an additional Cy5 label at the 5′-end of the siRNA antisense strand. However, no silencing activity was detectable due to trapping of the conjugates in endosomes. Therefore, a monodisperse polycationic carrier based on polyethylenimine-like 1,2-diaminoethane units [255] was applied to form polyplexes mediating endosomal escape via the proton sponge effect [256]. Silencing of GFP was then found to be dose-dependent and an 80% reduced expression at 25 nM was ascertained, whereas complexed non-conjugated siRNA or PEG-siRNA did not exhibit any effect at all. This proved the FolA-FR ligand-receptor pair to be suitable for the targeted delivery of ON via attachment of FolA as a small molecule targeting unit.

**Figure 4:**
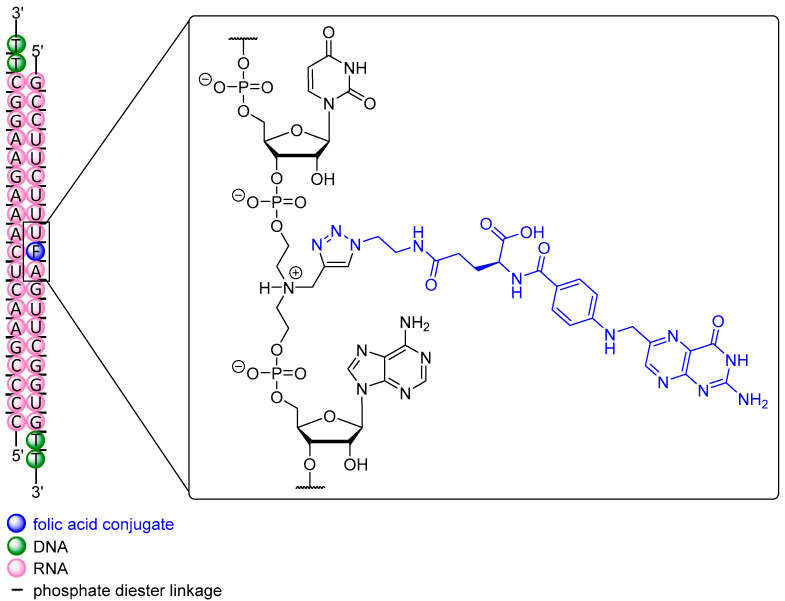
Internally modified folic acid-siRNA (passenger strand) for the silencing of Bcl-2 expression. The 5′-ends of siRNA strands are phosphorylated (not shown).

In analogy to the AEA-conjugate described above, Carell et al. [222] proved silencing in another reporter gene assay to be 50% at 1 μM using a FolA conjugate, with the ligand attached (by CuAAC) to the nucleobase at the 3′-end. However, in recently published work by Desaulniers et al., a remarkable conjugate was applied in HeLa and HT-29 cells with a FolA unit at several positions within an siRNA, representing the first FolA conjugate ever constructed in this fashion (Figure 4) [51]. An *N-*propargyl diethanolamine-derived unit was chosen as anchor point for subsequent FolA conjugation (via CuAAC) and inserted between two nucleotides at position 10, thus replacing a single nucleotide. This strategy also aimed at a destabilization of the central region of siRNA that spans the Ago2 cleavage site, thus leading to enhanced silencing activity [46,49]. Two conjugates with FolA-modified backbones were tested in a reporter gene assay in HeLa cells and showed a significant degree of silencing with IC_50_ values of 171 nM and 129 nM, respectively, after 24 h. Even at 1 nM, both FolA conjugates still exhibited 20 % inhibition whereas non-conjugated siRNA did not show any silencing at all. As anticipated, no silencing was observed in HT-29 cells for all siRNA targeting luciferase. HT-29 cells express 3-fold less FR, rendering them as a suitable FR-negative control. Compared to the previous work by Carell et al. in which FolA is installed at the 3′-end (IC_50_ = 1 μM), a 5-fold increase in silencing activity was achieved applying FolA-conjugation at the internal Ago2 cleavage site of the sequence [236]. The potential of this approach was also investigated with structures containing variable spacer lengths between backbone and ligand [52] as well as with cholesterol conjugates within the passenger strand [53]. However, the conjugate with internal modification was further tested in HeLa cells targeting endogenous Bcl-2, an anti-apoptotic protein that is overexpressed in several types of cancer [257,258]. At 1 μM, a reduction of 70% of Bcl-2 expression after 24 h was found, proving this extraordinary approach for a small molecule-ON conjugate to be a promising strategy for additional future studies.

### 2.4. Carbachol Conjugates

Due to its structural similarity to acetylcholine, carbachol is a parasympathomimetic drug that binds to G protein-coupled muscarinic (mAChR) as well as to nicotinic (nAChR) receptors [259]. Muscarinic receptors are targeted by small molecules for the treatment of neuronal disorders such as schizophrenia and Parkinson’s disease [260] as well as COPD [261].

Pauley et al. [262] exploited the ligand-receptor pair carbachol-muscarinic type 3 (M3R) to address Sjögren’s syndrome, a long-term inflammatory rheumatological auto-immune disease affecting moisture-producing glands, in particular the parotid gland. Affected patients suffer from loss of secretory functions of lacrimal glands and are at risk to develop lymphoma when the disease progresses [263]. The current therapy for Sjögren’s syndrome is based on secretagogues such as pilocarpine [264,265] to induce secretion via muscarinic receptor stimulation [266], mainly focusing on treating symptoms and lowering the risk of developing cancer [267]. However, it does not address the underlying causes of secretory dysfunction [262]. In the mentioned work, carbachol was post-synthetically conjugated to the 5′-end of a 19-mer siRNA sense strand via carbamate formation between a 5′-terminal amino group and activated choline (structure not shown). The siRNA was designed to target caspase-3 to prevent cytokine-induced apoptosis. Cytokines are main regulatory polypeptides of the innate and adaptive immune system and control all aspects of duration and modulation of the immune response [268]. In the case of Sjögren’s syndrome, however, an inherent imbalance of pro-inflammatory cytokines such as INF and TNF-α (overexpressed) and anti-inflammatory congeners such as TGF-β (low expression) leads to damage in glands, thus ultimately causing decreased secretory function [269]. To investigate the potential effect of the aforementioned siRNA, the synthesized conjugates were transfected into HSG cells that mimic Ca^2+^-signaling of salivary glands [270] resulting from receptor binding of carbachol. Measuring the expression levels of caspase-3 after 48 h revealed a reduction of 78% at 100 nM, whereas non-conjugated siRNA failed due to insufficient uptake. The latter was proven by calcium release assays and fluorescence experiments. Finding OAS-1 and MX-1 levels unchanged confirmed that no unspecific interferon response caused this downregulation of caspase-3. Such ON-small molecule conjugates therefore could be a starting point for a novel curative treatment for patients suffering from Sjögren’s syndrome instead of current symptom-oriented therapies.

### 2.5. Estrone Conjugates

To the best of our knowledge, there are very few examples of single or non-formulated ON directly conjugated to estrone (E_1_) applied in any therapeutic approach. Bang et al. synthesized two estrone-derived phosphoramidites (one bearing an octyl spacer) and attached them to the 5′-end of a 21-mer siRNA targeting vascular endothelial growth factors (VEGF) [271]. VEGF is an important signal protein family involved in the de novo formation of blood vessels (angiogenesis) as well as in lymphangiogenesis [272,273]. It is overexpressed in many types of cancer [274] as it is responsible for the permeability of blood vessels [275] and thus opens the blood-tumor barrier [276], ultimately causing metastasis [277] in particular in breast cancer [278]. E_1_ represents one of the natural endogenous female sex hormones besides estradiol and estriol [279] and acts as an agonist of the estrogen receptors alpha (ER-α) and beta (ER-β) [280]. Consequently, E_1_ conjugates were tested in ER-positive MCF-7 cells and showed a 3–4-fold enhanced silencing of VEGF expression as compared to non-conjugated siRNA (20%) without transfecting agents at 50 nM. However, nuclease stability remained unchanged, leading to 80% degradation after 24 h, suggesting that the enhanced activity resulted from improved uptake as also confirmed by confocal imaging. The conjugates equipped with an siRNA targeting lamin A/C mRNA [281] (that encodes a set of proteins being part of the inner nuclear membrane) were tested in ICR mice. Mutations in the LMNA genes further result in severe diseases, e.g., the Hutchinson-Gilford progeria syndrome [282] or several muscular dystrophies [283]. The results showed a clear downregulation of lamin A mRNA in a range of tissues, particularly in mammary glands by 60% at 40 nmol per mouse after 48 h, whereas non-conjugated siRNA did not exhibit effects in any tissue at all. Hence, exploiting ligand-receptor interactions by means of small molecule conjugation to ON again gave superior efficacy relative to the administration of standard siRNA for gene silencing.

**Scheme 1:**
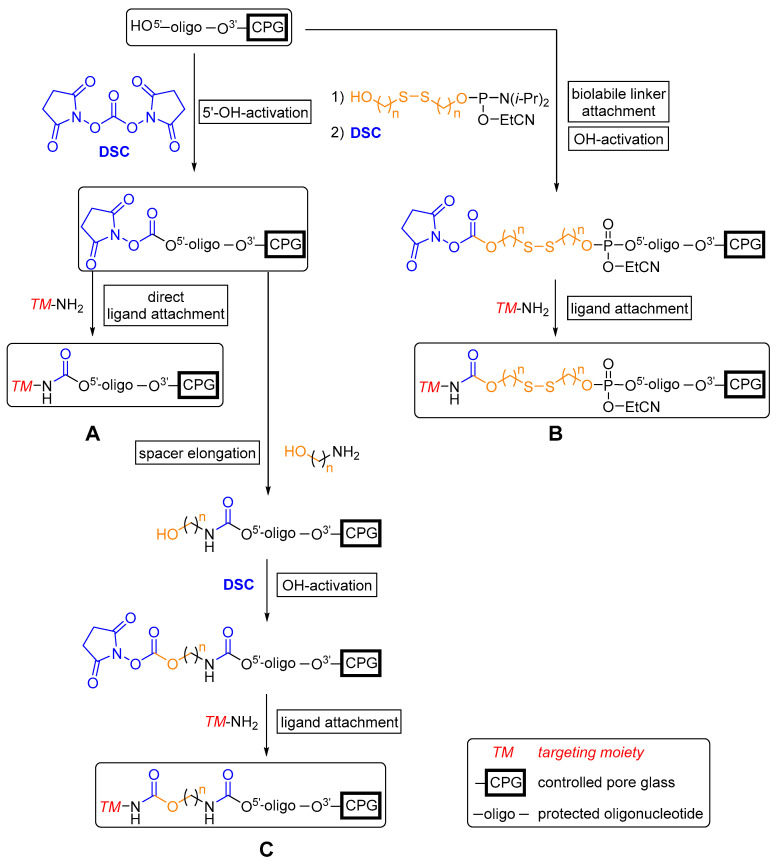
The potential of solid phase-supported synthesis for the preparation of 5′-conjugated ON based on carbamate formation (DSC = *N*,*N*’-disuccinimidyl carbonate). (**A**) Direct synthesis of 5′-functionalized ON. (**B**) Attachment of a biolabile linker with subsequent carbamate formation and attachment of the targeting moiety. (**C**) Spacer elongation via successive solid phase-supported reactions with final attachment of the targeting moiety.

Recently, Venyaminova et al. [284] reported the development of a new solid phase-supported approach to attach several functional amino-modified ligands, in particular estrone, to the 5′-end of protected (dT)_7_ ON model sequences by *N*,*N*’-disuccinimidyl carbonate (DSC)-mediated carbamate formation (Scheme 1). This strategy enabled variations in the length and size of spacer units, e.g., hexamethylenediamine, between an ON and (multiple) amino-modified ligand(s) by several successive reactions. Employing this methodology can overcome issues correlated with post-synthetic approaches such as the search for a suitable solvent (mixture) for post-synthetic modifications with highly hydrophobic entities. Furthermore, the often moderately yielding syntheses of phosphoramidite derivatives of effector molecules is rendered obsolete. A straightforward introduction of an amino group in a position that does not impede receptor interaction is thus sufficient for the conjugation of small molecules with therapeutic ON on resin.

It also should be noted that a conjugate with an estrone-appended polyion micelle to deliver a complexed cytotoxic peptide to breast cancer cells was recently reported [285]. Targeting ER-α-related diseases is attracting growing interest, in particular in the case of hormone-responsive types of breast cancer. Readily prepared ON conjugates with small molecules to enable antisense-mediated gene knockdown represent a promising option to further exploit ER binding for therapeutic purposes.

### 2.6. GalNAc Conjugates

The main strategy for inhibiting genes expressed in hepatocytes involves the exploitation of the asialoglycoprotein (Ashwell-Morell) receptor that is highly expressed in hepatocytes [286]. Upon binding, glycoproteins terminated with *N*-acetylgalactosamine (GalNAc) are readily internalized via clathrin-mediated endocytosis [287]. The discovery that this receptor can be exploited for tailor-made drug delivery [288], even for therapeutic ON [289], paved the way for extensive research on delivery formulations [290] to address targets previously classified as ‘undruggable’. These included, for instance, genes of hepatitis C virus (HCV) [291] or lipoprotein A [292] that is correlated with heart attacks and strokes. Nowadays, many GalNAc conjugates are known [293,294,295,296] and some recent examples are discussed here [54,297,298,299], in particular multivalent GalNAc motifs that proved to have superior targeting efficiency [300,301] when combined with established ON backbone modifications [302].

In rather recent work [299], multivalent di- [(GalNAc)_2_] and triantennary [(GalNAc)_3_] GalNAc units were installed via a *trans*-4-hydroxyprolinol linker to a solid support to yield a set of 3′-end (sense strand) modified 21-mer siRNA with PS backbone at 3′-ends and 2′-fluoro-, 2′-OH- and 2′-*O*-methyl motifs within the strands. Cellular uptake of these conjugates was tested at 20 nM in freshly isolated primary mouse hepatocytes [303] using a fluorescence-based assay. A comparison between triantennary and biantennary GalNAc conjugates in vitro revealed a correlation of ligand binding affinity and increased uptake, with the latter being 8-fold higher for (GalNAc)_3_ and 3-fold higher for (GalNAc)_2_ relative to non-conjugated siRNA or a biantennary conjugate with glucose (as negative control). Treatment of hepatocytes gave efficient and rapid receptor cycling as expected based on previous literature [287], thus further proving a receptor-mediated process. The (GalNAc)_3_ conjugate was additionally tested for silencing of the rodent transthyretin (TTR) gene in vivo in C57BL/6 mice and showed 80% reduction of TTR expression with a single dose of 5 mg/kg. A weekly dose of 2.5 mg/kg rendered TTR expression levels below 20% for over nine months, demonstrating that the conjugate was extremely effective upon subcutaneous injection that was superior to intravenous application. Mutated TTR genes cause hereditary transthyretin-mediated amyloidosis [304,305], a rare neurodegenerative disease [306,307] leading to paresthesia, muscular weakness [308] and autonomic dysfunction evoked by deposition of abnormal transthyretin in the peripheral nervous system [309,310].

**Figure 5:**
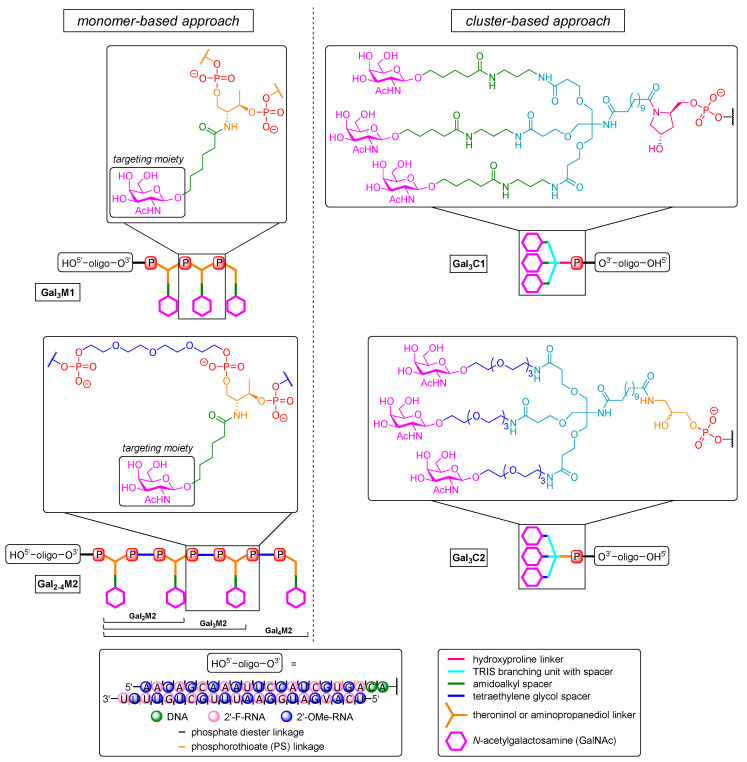
Overview of GalNAc conjugate structures targeting endogenous cyclophilin B. (**Left**) ‘monomer-based’ Gal_3_M1 and Gal_2–4_M2 are clusters constructed by successively reacted GalNAc-derived phosphoramidites with (M2) and without (M1) TEG spacer. (**Right**) the ‘cluster-based’ novel Gal_3_C2 unit (aminopropanediol-based) in comparison to the clinically studied Gal_3_C1 motif (prolinol-based) [321].

Khvorova, Watts et al. demonstrated the two main approaches by which GalNAc motifs might be linked to therapeutic ON (Figure 5) [298]. The first strategy, termed ‘cluster-based approach’, follows the design principle of the aforementioned trivalent structure that is also used in the controversial drug candidate Revusiran [311,312] (Gal_3_C1, Figure 5). Furthermore, it comprises an aminopropanediol-linked GalNAc dendrimer for 3′-end triantennary conjugates (Gal_3_C2, Figure 5). The acyclic aminopropanediol linker has also been used with tetraethylene glycol spacers instead of amidoalkyl units in order to reduce hydrophobicity. Such multivalent dendrimers could also be installed post-synthetically via solution-phase chemistry at both ends of the ON. The second strategy constructs GalNAc cluster structures by multiple couplings of GalNAc-derived phosphoramidites (to furnish up to four repeating units) and is termed ‘monomer-based approach’. The phosphoramidite monomer is based on l-threoninol with an amidoalkyl linker to the GalNAc unit (Gal_3_M1, Figure 5). The successively introduced targeting units can also be further separated by tetraethylene glycol spacers (Gal_2-4_M2, Figure 5) to ensure optimal topology for binding to the heterooligomeric receptor [301]. However, both monomer-based structures were installed on solid support to afford 3′-end conjugates at the sense strand of a 22-mer siRNA that was further modified (i.e., 2′-*O*-methyl, 2′-fluoro, partial PS backbone). Cy3 was additionally introduced as a fluorophore at the 5′-end of the sense strand to monitor biodistribution. The new dendritic trivalent GalNAc conjugate and the novel linear tetravalent conjugate showed higher hepatocyte binding in FVBN/J mice than a comparable conjugate that already is in clinical trials [299,313]. Gal_3_C2 gave 2-fold, Gal_3_M1 1.5-fold, and Gal_4_M2 even 3-fold increased association compared to Gal_3_C1 [299], and the same was found for liver accumulation. To investigate the silencing efficacy, structures were applied that targeted endogenous cyclophilin B (PPIB) [314]. The inhibition of cyclophilin proved to be effective for suppressing the replication of HCV [315,316,317]. All dendritic trivalent conjugates showed dose-dependent silencing of PPIB mRNA expression that was slightly better than for linear Gal_4_M2 (~60% at a dose of 2.5 mg/kg, 80% at 10 mg/kg after 48 h), representing a 2-fold increased potency relative to non-targeted controls. They were also recovered from the liver in 2-fold higher amounts than their linear counterparts. Of the linear multivalent conjugates Gal_2_M2, Gal_3_M2, and Gal_4_M2, the latter exhibited a stronger silencing effect and higher accumulation in the liver. This outcome observed for a tetravalent targeting moiety attached to siRNA is in some contrast to previous results for ASO [318,319], for which bivalent targeting moieties exhibited at least equal silencing than trivalent derivatives. This might demonstrate that the topology of such targeting units can be more relevant than valency for efficient receptor binding [320] with small molecules.

In other rather recent work, a unique dual cholesterol-GalNAc conjugate was used to lower nephrotoxicity [322] related to a certain class of ASO therapeutics (SPC5001) [323,324] by altered renal distribution with retained liver targeting. Cholesterol conjugation is known to extend the half-life of ON in blood, which leads to decreased kidney accumulation [325] and (as for GalNAc derivatives) enables liver targeting [326,327], but in this case, in non-parenchymal cells [328]. Wada et al. [297] combined the aforementioned 13-mer PS ASO (SPC5001) [329] with cholesterol as well as GalNAc units. This PS ASO construct targeted ApoB mRNA [329] to treat homozygous familial hypercholesterolemia [330] and contained deoxyribose nucleotides flanked by LNA moieties at both ends. The 5′-modification consisted of a linear arrangement of three *trans*-4-hydroxyprolinol phosphoramidites [331], each linked to a single GalNAc unit via an amidoalkyl linker. Cholesterol was attached either to the 5′-end via a tetraethylene glycole spacer (‘5GN-5Chol’) or to the 3′-end in the same manner (‘5GN-3-Chol’). GalNAc units as well as cholesterol building blocks were connected to the ON via phosphate diester linkages to enable the formation of the free ASO for high activity after cellular uptake [332,333]. The conjugates were tested in male C57BL/6J mice and gave rather astonishing results. Gene silencing and cholesterol clearance exhibited by the dual conjugates 5GN-5Chol and 5GN-3Chol were found to be only slightly lower at a dose of 8.75 nmol/kg (~80% vs. 70% knockdown), indicating no significant benefit relative to the original GalNAc conjugate (‘5GN’). On the other hand, renal uptake of the dual conjugates was reduced by one-fifth compared to 5GN and by one-fiftieth compared to the non-conjugated ASO. This is likely due to a transport mechanism mediated by cholesterol [325], i.e., binding to high-density lipoprotein as well as albumin [334], enhancing delivery to the liver [326]. Thus, exploiting the target preference of cholesterol for non-parenchymal cells [328] altered the biodistribution of the GalNAc conjugate that then exhibited inherent preference for hepatic tissue [319] towards reduced renal accumulation.

In recently published work [54], the influence of a single altritol hexopyranose modification (ANA) on the silencing potency of a 21-mer siRNA targeting rodent TTr mRNA was evaluated. This therapeutic ON (Patisiran) represents the first FDA-approved siRNA with activity against hereditary transthyretin-mediated amyloidosis [57] and was granted breakthrough status due to previous lack of an according drug. For potential further improvement, ANA was inserted once at each position in both the sense and the antisense strand. The resultant PS siRNA were further modified with 2′-fluoro and 2′-*O*-methyl units and conjugated to a trivalent GalNAc motif with a *trans*-4-hydroxyprolinol linker at the 5′-end. The GalNAc moiety was introduced by conventional phosphate diester formation, enabling release of the parent drug for optimal mRNA binding. As there are no naturally occurring enzymes for the cleavage of ANA, exo- and endonuclease-mediated degradation was blocked depending on the position of the ANA unit within the strands. Furthermore, the ANA-modification provides duplex stabilization via its hydroxyl group [335]. The conjugates were tested in primary mouse hepatocytes. It was found that placing the ANA unit at one of the 5′-ends furnished a 3-fold decrease in silencing activity in vitro relative to the parent drug. One explanation might be the impossibility for nucleoside kinases to phosphorylate the modified 5′-end [336], a process that is essential for the interaction with Ago2 [337] as part of RISC formation [338]. Placing the ANA unit in the seed region of the sense strand (positions 6 and 7) afforded nearly identical silencing of TTr mRNA as with the parent drug (95% vs. 98% degradation at 10 nM), as it was the case for the modification of position 16 (95% degradation). Further studies regarding the stability of (dT)_19_ with an ANA-modification at either end against degradation mediated by phosphodiesterase II (PDE II) or snake venom phosphodiesterase (SVPD), respectively, revealed strongly increased stability only against PDE II (90% vs. 40% retained full-length ON after 24 h). Thus, using the ANA-modification in combination with GalNAc conjugation can add enhanced 5′-exonucleolytic stability to potent small-molecule conjugates of therapeutic ON.

## 3. Conjugation of ON with Small Molecules for Enhanced Endosomal Release

### Conjugates with Retro-1

The molecule Retro-1 became a candidate for conjugation based on a high-throughput screening of small molecules to block the effects of bacterial toxins such as shiga [339] or ricin [340] via inhibition of retrograde toxin transport between early endosomes and the TGN/Golgi complex [191]. This furnishes the connection to ON delivery: internalization of ON via any form of endocytosis is followed by participation in cellular traffic of the therapeutic. Therefore, ON have to pass through numerous endomembrane compartments [152], leading to a sequestration of most of them in endosomes, thus ultimately weakening the biological effect due to a reduced abundance in the cytosol [341]. It was shown that besides other small molecules [342], Retro-1 can mediate the aforementioned effect and therefore improves the redistribution of SSO and ASO [343] to the cytosol, leading to an increased concentration of ON in the nucleus [344]. However, it does not improve the distribution of siRNA. Retro-1 blocks the retrograde trafficking pathway as it interacts with shuttling processes, hence causing rapid partial release of ON from a subset of endomembrane compartments [343].

**Figure 6:**
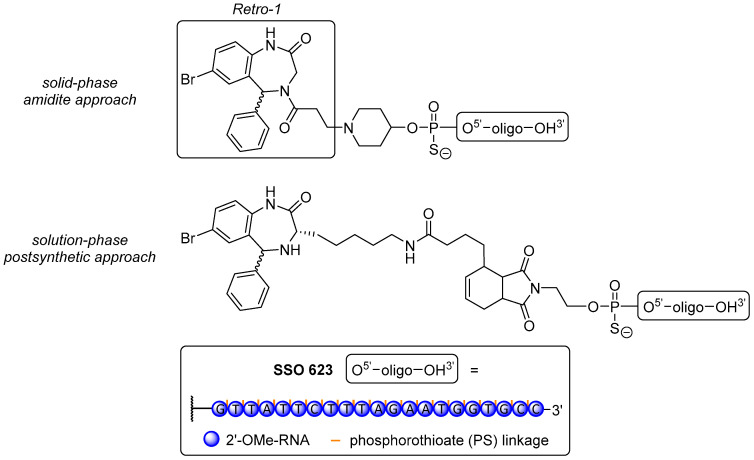
Retro-1 conjugates with the splice-switching ON SSO 623. (**Top**) on-resin conjugation product of Retro-1 with the ON using a 4-hydroxypiperidine-phosphoramidite. (**Bottom**) postsynthetic conjugation product of a Retro-1 derivative with the SSO via Diels-Alder reaction.

Juliano et al. [345] prepared three Retro-1 derivatives, covalently attached to the 5′-end of a 20-mer PS SSO with 2′-*O*-methyl modifications via conventional amidite coupling on resin, or via Michael-type click reaction and Diels-Alder cycloadditon (post-synthetic, solution-phase reactions, Figure 6). The conjugates were tested in a luciferase reporter gene assay with the well-known splice switching ON sequence SSO 623 [176] in HeLa Luc 704 cells. Unfortunately, none of the novel conjugates afforded an improvement in splice-switching activity over the dual use of SSO 623 with UNC7938 as a widely used endosome-destabilizing agent [344,346] or even SSO 623 alone. The reasons for this have not been elucidated yet, but ongoing research attempts to use more potent analogues of Retro-1 [344,347] or variations of spacer units to overcome possible impairments with the splice-switching machinery.

## 4. Conjugation of ON with Lipophilic Small Molecules for Enhanced Membrane Penetration

The attachment of lipophilic small molecules to ON (to give ‘LON’) is a proven approach to enhance both (specific) cellular uptake [334,347] and endosomal release of the resultant conjugate [348]. The penetration of biological membranes by highly charged polar ON sequences is naturally hindered. This limits the overall activity of such therapeutic agents in vivo due to hampered cellular uptake (which is dependent on a plethora of pathways) and limited release from endocytotic vesicles. To overcome these intrinsic challenges, two major issues have to be addressed: poor cellular delivery and poor escape to the cytosol. The former can be improved by attaching lipophilic moieties to the ON to take advantage of the endogenous lipoprotein transport machinery. Thus, low-density lipoprotein (LDL) [349] and high-density lipoprotein (HDL) [350] mediate transport and internalization of lipids such as cholesterol. Furthermore, ON (in particular PS ASO) also interact with plasma proteins and/or lipoproteins to enter hepatocytes via receptor-mediated or non-mediated processes [326,351,352]. Hence, it was demonstrated that LDL and HDL facilitate internalization of lipid-ON conjugates [353]. In another study, conjugated ASO preincubated with lipoproteins were shown to have a lower effect in LDLR-knockout mice than in wild-type animals [354], further highlighting the benefit resulting from attached lipid small molecules.

With respect to endosomal release, exogenous ON have to participate in intracellular traffic and take advantage of the multitude of fusion and disjunction events to which vesicles are subjected in order to escape into the cytosol [355,356]. Lipid-ON conjugates bound to LDL [327] are mainly internalized via the clathrin pathway [357,358] and thus undergo four common steps of downstream traffic after receptor binding: (i) pinching-off from the donor compartment (cell membrane with specific receptors binding the lipid); (ii) vesicle uncoating; (iii) actin/tubulin-mediated transport from the donor (cell surface) to the recipient (early endosomes); and (iv) SNARE protein-assisted fusion of both compartments to ultimately deliver the conjugate to its destination [359]. Within this pathway, a rather simple effect opens up the possibility to escape the aforementioned machinery and enter the cytosol. During pinching-off and fusion events, the formation of non-bilayer vesicles is observed [360,361] that tend to be leakier, hence providing an inherent opportunity for endosomal escape in particular for lipid-conjugated ON [362]. Crooke et al. demonstrated additional membrane destabilization due to lipids, thus increasing endosomal leakage [363]. Further enhanced protein and membrane binding enables in particular LON to remain bound to membranes controlling endosomal release [364]. Some previous and current work on LON is discussed in this section.

### 4.1. Conjugates with Cholesterol

Among lipophilized ON, those modified with cholesterol represent by far the most abundant sub-group of conjugates and have therefore been reviewed elsewhere in detail [334,347,352,365,366]. Within the scope of this review, only some selected prominent or recent examples are presented in detail.

Soutschek et al. [367] have reported the first pioneering in vivo proof-of-concept for triggering RNAi with siRNA. They chose a 21/23-mer siRNA to target endogenous ApoB mRNA and silenced its expression and as a result total levels of cholesterol—with a conjugate bearing a cholesterol unit at its 3′-end. Cholesterol was installed via a pyrrolidine-derived linker on the solid support at the sense strand, with phosphorothioate linkages and two 2’-*O*-methyl nucleotides incorporated to ensure stability against exonuclease-mediated degradation (structure not shown). In C57BL/6 mice, the resultant construct decreased levels of ApoB-100 mRNA by 57% in the liver and 73% in the jejunum. ApoB-100 plasma levels were found to be decreased by 68% after 24 h, and the total amount of cholesterol was shown to be reduced by 40% at a dose of 50 mg/kg. One explanation for this increased biological activity relative to non-conjugated ON might be the fact that the conjugate interacts with (serum) albumin or lipoproteins (LDL and HDL) to enter hepatocytes via their specific receptors, leading to some sort of cellular targeting [326,351,352]. This work has stimulated further research on the site-specific delivery of ON for the silencing of ‘undruggable’ targets.

**Figure 7:**
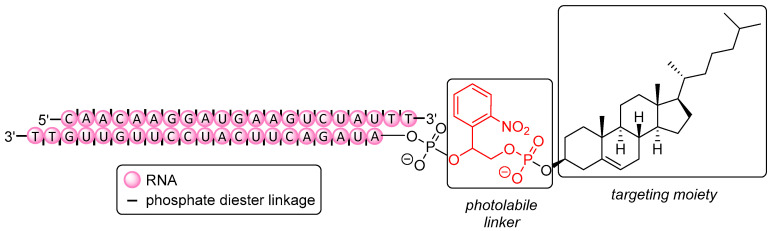
Cholesterol-modified caged siRNA targeting mitotic kinesin-5 (Eg5) mRNA.

Tang et al. [368] have described the conditional regulation of gene expression via spatiotemporal control of silencing activity with light as a non-invasive trigger (Figure 7). Therapeutic activity of small molecules [369] or ON [370] can be blocked (‘caged’) with photolabile moieties (PL), e.g., NPE [371], NPP [372], NPOM [373], and DMNPE [374]. In the case of ON, the photolabile units render them unable for hybridization when such units are attached to nucleobases, thus disturbing Watson-Crick base pairing. On the one hand, ON are thus more protected against immune response or exonuclease degradation [375,376], and on the other hand, they do not exhibit any premature silencing activity leading to off-target effects. As shown by Tang et al., attaching such a PL together with cholesterol to the 5′-end of the antisense strand of a 21-mer siRNA designed for downregulation of the Ago2 protein (Figure 7) resulted in failure to form the RISC [377]. However, photo-induced release (λ = 365 nm) of native siRNA took place within just a few minutes, rendering the ON active again. Photomodulation of gene expression was also proven by effective silencing (after irradiation) of mitotic kinesin-5 (Eg5) mRNA (that is crucial for mitosis [378,379]) using a photolabile conjugate. Compared to a positive control at 5 nM (non-caged siRNA), Eg5 mRNA expression was slightly less reduced using the conjugate (80% vs. 60%), but the total amount of cells arrested in the G2/M phase was nearly the same (40%), showing the photo-activatable conjugate to be similarly potent as the parent drug.

In contrast to the attachment of small molecules either to the 5′- or to the 3′-end of ON, there are few approaches introducing cholesterol within an ON sequence via modified nucleoside building blocks. These have the cholesterol unit either connected to the nucleobase [55] or at the 2’-*N*-position in the case of amino-LNA [380], using conventional phosphoramidite chemistry for ON formation in both cases. In an early example, Letsinger et al. [381] used oxidative phosphoramidation on resin to attach amino-modified cholesterol via H-phosphonate chemistry to an internucleoside phosphorus. One can also choose non-nucleosidic cholesterol derivatives [382] as well as non-nucleosidic linkers designed for post-synthetic derivatization with cholesterol via a plethora of reaction types [365]. In a recent study, Nakajima et al. synthesized an ON conjugate having a cholesterol thiono triester within the phosphate backbone (structure not shown) and evaluated delivery and silencing activity relative to alternative cholesterol attachment to the 5′- and 3′-ends for the first time [56]. A set of 13-mer PS ASO and phosphate-linked (PO) ASO designed to silence ApoB expression [329] was synthesized with LNA units at both ends to enhance stability against nucleases. Within the structure, a modified thymidine-derived (T) phosphoramidite was inserted at several positions (4, 8, and 10) with a triethylene glycol-derived cholesterol attached to the phosphates. In general, adequate space between the ON and the small molecule as well as ideal placement of the latter are very important factors to be considered in the design of potent conjugates, in particular when the ligand is placed at the 5′- or 3′-end [56,332]. The delivery of 5′(PS), 5′(PO), and T10 (PS) ASO conjugates in C57BL/6 mice at 0.5 mg/kg was strongly enhanced (15-fold, 3-fold, and 10-fold, respectively). In contrast, the silencing efficacy of the conjugates remained insufficient except for the 5′(PO)-linked derivative (5-fold increase), all relative to non-conjugated ASO. These findings indicate that a rapid cleavage of the small molecule from the ON scaffold might be potentially mandatory in order to achieve increased silencing alongside improved delivery [328].

### 4.2. Conjugates with α-Tocopherol

Several studies have been reported in which therapeutic ON were conjugated with α-tocopherol (α-TP) for the purpose of efficient gene silencing [354,383,384,385,386,387]. α-TP is one out of four tocopherol congeners (α, β, γ, δ) that show inherent antioxidant [388], anticancer [389], anti-inflammatory [390], immuno-stimulatory [391], and nephroprotective properties [392,393]. Together with the three-fold unsaturated analogues, i.e., the four tocotrienols, they form the vitamin E family, a major bioactive class of essential nutrients for human metabolism [394,395]. Tocopherols are therefore considered to be effective ligands for drug delivery of poorly internalized ON therapeutics, also with respect to their active protein-mediated uptake [396,397], as described by Nishina et al. with the first α-TP conjugate. A 27/29-mer siRNA [383] having α-TP connected to the 5′-end of its antisense strand as well as several standard modifications (2′-*O-*methyl, partial PS backbone; structure not shown) was designed to reduce expression of endogenous ApoB in the liver, in order to reduce cardiovascular events after the dicer-assisted formation [398] of mature 21-mer siRNA including cleavage of the α-TP targeting unit. The stability of the conjugate in mouse serum was improved 4-fold as compared to the non-conjugated siRNA (that was fully degraded after 8 h). As shown via confocal imaging, the α-TP conjugate was readily taken up by the liver, thus delivering the 27/29-mer as well as the mature 21-mer siRNA, whereas uptake of non-conjugated siRNA could not be found. Levels of endogenous ApoB-encoding mRNA in the liver were evaluated 48 h after administration of the conjugate in C57BL/6 mice and were decreased by 50%, whereas no silencing with non-conjugated reference siRNA was observed at a dose of 2 mg/kg.

Further work reported by the same group [384] showed a strong increase in delivery of a 5′-α-TP-modified 27-mer siRNA (2′-*O-*methyl, partial PS backbone; structure not shown) to neurons, particularly in the cerebral cortex and hippocampus, at low doses. After intracerebroventricular (ICV) administration in female Crlj:CD1 mice, non-conjugated siRNA could not elicit detectable target gene silencing, whereas an amount of 100 nmol/kg of the conjugate furnished 70% decrease of beta-site amyloid precursor protein cleaving enzyme 1 (BACE1) mRNA after seven days. A comparison with similar gene silencing trials [399,400] highlighted the reduction of the required dose of siRNA by three orders of magnitude. The additional use of high-density lipoprotein (HDL) as transfecting agent ameliorated receptor-mediated uptake via HDL receptors [397,401]. This might also have an impact on the development of drugs against Alzheimer’s disease [402,403]. In general, conjugated ASO preincubated with lipoproteins such as HDL showed lower effects in LDL-knockout mice than in wild-type animals, thus further proving receptor-mediated uptake exploiting this pathway [354].

Nishina et al. [354] reported the construction of a highly effective heteroduplex ON (HDO) consisting of a DNA/LNA-gapmer as a 13-mer PS ASO and a 31-mer cRNA complementary to this PS ASO, with the latter having an α-TP unit at its 5′-end and some 2′-*O*-methyl modifications (structure not shown). This unique structure was applied to improve the delivery of the ASO via hybridization with the sense strand of an α-TP-conjugated cRNA to evaluate silencing of ApoB mRNA [329] and of scavenger receptor class b member 1 (Srb1) mRNA [404] in mice. The α-TP-HDO was found to be 22-fold more potent (ED_50_ = 38 μg/kg) than the sole PS ASO (ED_50_ = 841 μg/kg) for the downregulation of ApoB expression in the liver. Three days after administration of 0.75 mg/kg to C57BL/6 mice, ApoB mRNA levels were reduced by 50% and 40% using the PS ASO and the non-conjugated HDO, respectively. In contrast, a reduction of more than 90% of ApoB mRNA levels was observed for the α-TP-conjugated HDO, while the employed cRNA alone did not exhibit any effect. The amount of α-TP-HDO delivered to the liver was 5-fold higher relative to the non-conjugated HDO or the PS ASO alone. With respect to duration of ApoB mRNA silencing, the non-conjugated HDO exerted only 10% reduction after 14 days, whereas the α-TP-conjugated HDO furnished 70% reduction even after 28 days. Srb1 levels in mice were reduced by 60% using the PS ASO alone at 10 mg/kg. In contrast, the α-TP-HDO conjugate afforded a reduction of Srb1 levels of more than 90% at the same dose. No detectable increase of IFN-γ or TNF-α levels in any of these experiments indicated that no undesired immune-stimulatory effect had occurred. Using the same mouse model, Nishina et al. [385] have also evaluated the effect that several LNA-modifications have on the silencing efficacy towards ApoB1 and Srb1.

A comparative study [328] investigated the dose, efficacy, and duration of a trivalent GalNAc-, a cholesterol- and a 5′-α-TP-conjugated 13-mer ASO [329] (all linkages PS-modified, LNA units at both ends; structures not shown). All three resultant conjugates targeted the same ApoB1 mRNA in hepatocytes as well as in non-parenchymal cells in male C57BL/6 mice. The α-TP and cholesterol moieties were installed via conventional phosphoramidite chemistry on resin. The GalNAc unit was introduced post-synthetically via carbamate formation with an aminohexyl linker. At a dose of 1 mg/kg and relative to the non-conjugated ASO, all of these ASO conjugates exhibited multifold improved delivery of the ASO to the liver. However, only the cholesterol-ASO and the α-TP-ASO showed higher delivery in non-parenchymal cells. In general, ASO tend to accumulate preferentially in non-parenchymal cells, but they are intrinsically more active in hepatocytes [405]. Similar results were obtained regarding the efficacy of ApoB mRNA silencing in both the liver as a whole and in hepatocytes, with the GalNAc conjugate being superior to the other congeners with the exception in non-parenchymal cells, in which the α-TP conjugate showed the highest silencing efficacy. All conjugates displayed an extended duration of the silencing effect up to several days after injection while their half-lives were similar to the non-conjugated ASO. Overall, these results might provide options for a more efficient ON-mediated silencing of oncogenes in different types of liver cells, with conjugated small molecules steering the biological effect.

**Figure 8:**
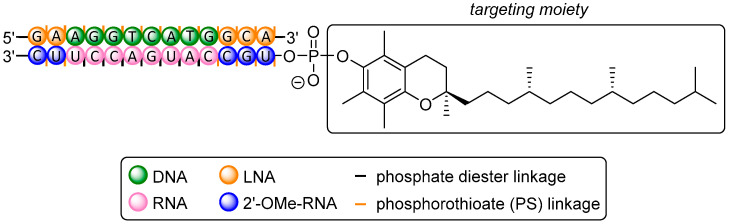
Structure of the α-TP-HDO conjugate designed to target the expression of OAT3 in BEMEC.

In a recent proof-of-concept study, a new potential platform technology for targeting intractable neurological diseases has been described [386]. The aim of this work was to modulate the properties of the blood-brain barrier (BBB) with ON. Thus, an HDO conjugate was designed to target the expression of organic transporter 3 (OAT3) in brain microvascular endothelial cells (BEMEC). OAT3 is almost exclusively expressed in BEMEC and represents an interesting drug target for several reasons. First, protocols exist for the quantification of biological activity of OAT3 with respect to its expression pattern within the BBB [406]. Second, no major pathological side effects are expected as demonstrated with *oat3*-knockout mice [407]. A PS ASO flanked by several LNA-modifications at both ends was hybridized with complementary 5′-α-TP-cRNA (PS- and 2′-*O*-methyl-modified) to yield a potent HDO (Figure 8) [354]. This was used to target exogenous luciferase-fused OAT3 genes pre-transfected to C57BL/6 mice. Two of these model genes of OAT3 were downregulated by 86% and 50%, respectively, at a dose of 32 mg/kg without the use of a transfecting agent. However, the silencing of one gene construct was accompanied with severe liver dysfunction and induction of IFα-γ and TNF-α in these mice. These observations were also made though when the sole ASO without conjugated α-TP unit was administered. Nevertheless, this work has demonstrated an interesting approach to address neurological disorders via the use of a small molecule-conjugated ON. This strategy might potentially contribute to improved therapies of diseases such as, for instance, multiple sclerosis [408] or Alzheimer’s disease [409].

### 4.3. Conjugates with Squalene

Squalene (SQ) is an endogenous triterpene, produced in the liver and skin as precursor in the biosynthesis of steroids such as cholesterol [410,411]. Due to its high hydrophobicity, conjugation of squalene to the 5′- or 3′-end of an ON furnishes a highly amphiphilic construct tending to self-aggregate to nanostructures, ultimately leading to increased plasma stability without the need for formulating agents. This kind of self-aggregation might save the potential patient from undesired side effects related to the toxicity of common formulations [412,413] or a burst release of the drug from the formulation [414]. The concept of squalenylation has already been used in the context of drug delivery [415,416,417,418,419] since its first application to improve the delivery of gemcitabine, a cytotoxic nucleoside analogue used in anticancer chemotherapy [420].

Raouane et al. [421] attached the triterpene to an ON via Michael-type click reaction between a maleimide-modified squalene and a mercaptopropylphosphate residue at the 3′-end (sense strand) of a 19-mer siRNA [422] targeting RET/PTC1 (reaction not shown). The latter represents an oncogene found in papillary thyroid carcinoma [423] as it encodes an abnormal membrane tyrosine kinase [424]. The conjugate was tested in TPC-1 and in BHP 10-3 cells and did not show any growth inhibition or significant toxicity in vitro. However, in vivo testing of the SQ conjugate in nude mice revealed growth inhibition in xenocrafted tumors of 70% after injection of a 2.5 mg/kg dose. RET/PTC1 mRNA expression was reduced by 80% after 24 h whereas non-squalenylated siRNA displayed only 10% inhibition additionally using Lipofectamine 2000 as transfecting agent.

Urbinati et al. [425] constructed an SQ conjugate based on a 21-mer siRNA to silence the oncogene TMPRSS2-ERG [426] that is found in 50% of prostate-related cancers [427]. This new approach is aimed to bypass toxicity issues [428] of hormone-dependent prostate cancer treatment with flutamide [429], the standard in nonsteroidal antiandrogen therapy that thus provokes discontinuous treatment. Targeting the fused gene [430] consisting of TMPRSS2 [431] and ERG [432] that encodes ETS transcription factors [433] might also be a salvation for patients who suffer from androgen-independent [434] or castration-resistance prostate cancer [435,436]. Based on previous in vitro studies [437,438], the SQ conjugate could only be applied with the aid of transfecting agents to cause tumor growth inhibition of 70% at 50 nM. However, uptake was ascertained without transfecting agents in vivo, evoking a 3-fold weaker tumor growth relative to non-conjugated siRNA at a cumulative dose of 1.8 mg/kg after 40 days. A reduction of 60% in mRNA levels of TMPRSS2-ERG oncogenes (REG4 and PHGR1) was found at 50 nM after 72 h, while non-conjugated siRNA did not exhibit any decrease at all. Overall, these results demonstrate that SQ conjugates can be therapeutically very effective, but that disparities between in vitro and in vivo activities may occur.

### 4.4. Conjugates with Fatty Acids

Besides other hydrophobic small molecules, unbranched fatty acids can also be attached to therapeutic ON. For instance, saturated congeners such as lauroyl (C_12_), myristoyl (C_14_), palmitoyl (C_16_), stearoyl (C_18_), and docosanoyl (C_22_) units as well as (poly-)unsaturated structures such as oleoyl (C_18_, ω-9) and linolenoyl (C_18_, ω-6,9) were conjugated to a 21-mer siRNA for in vitro targeting of ApoB mRNA [327]. With respect to their similarity to cholesterol, conjugates with bile acids are not further discussed within this review, but have been reviewed elsewhere [439,440,441].

The probably most relevant example of a fatty acid conjugate is Imetelstat (GRN 163L) that is currently in clinical trials phase II (trial reference NCT01731951) [442]. Imetelstat is a 13-mer thio-phosphoramidate (N3′→P5′) ASO that is modified at its 5′-end with a palmitic acid unit, attached via an aminoglycol linker (structure not shown). This drug candidate is intended to target human telomerase (hTR) for the treatment of myelofibrotic cancer [443]. Telomere elongation is frequently observed in cancer cells [444], rendering them ‘immortal’ due to maintained cell division or the lack of cell cycle arrest, while healthy cells undergo progressive telomere shortening upon repeated cell division [445]. To overcome this intrinsic property of cancerous cells, Herbert et al. chose palmitic acid as a small-molecule modifier to improve the poor delivery of the known ASO sequence [446] and to enhance its availability in target tissue. Assays in HME50-5E, PC3 (prostate cancer), and MDA-MB231 (breast cancer) cells revealed reduction of telomerase activity at 1 μM by 73%, 93%, and 69%, respectively, representing a nearly 40-fold increase [447] in activity relative to the non-conjugated ASO due to optimized pharmacokinetics.

More recently, plasma protein binding of palmitoyl, cholesterol, and tocopherol conjugates for the improved silencing of MALAT1 in rodent muscles has been studied [387]. Targeting skeletal muscle cells at the genetic level is a novel approach for the treatment of Duchenne muscular dystrophy [448]. The ASO drugs Eteplirsen [36] and Golodirsen [34] were developed to achieve this goal, but their success was limited [449]. MALAT1 was proven to also play a key role in metastasis of carcinomas [450] in mouse models and therefore represents a promising target for additional research. Østergaard et al. have investigated a 19-mer ASO (partial PS backbone, cEt-modified at several positions; structure not shown) conjugated to palmitic acid via an amidohexyl phosphoramidite in C57BL/6 mice and cynomolgus monkeys [387]. They have found stronger binding of all conjugates to albumin and additional binding to LDL and HDL as compared with the non-conjugated ASO, indicating a potentially improved delivery to target tissues. Rodents and monkeys were treated with a cumulative dose of 40 mg/kg over seven weeks, and expression levels of dystrophiamyotonica protein kinase (DMPK) mRNA were found to be 2- to 3-fold lower in several muscle tissues without any notable toxicity when α-TP- or palmitoyl-conjugated ASO were applied. A cholesterol-conjugated ASO exhibited slightly higher toxicity in rodents and was therefore not tested in monkeys. Thus, the exploitation of plasma protein binding with fatty acid small molecules to enhance the potency of known ASO sequences demonstrated the effectiveness of this approach.

**Figure 9:**
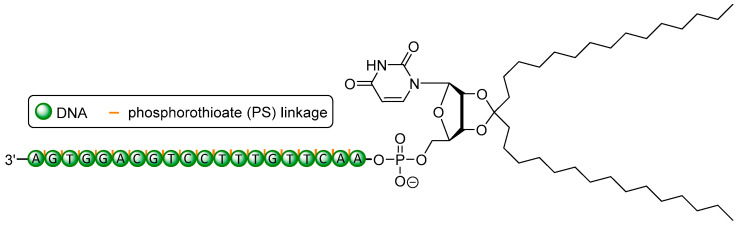
Structure of the ketal (2′,3′-*O*-(16-hentriacontanylidene)) nucleolipid-conjugated ASO for the silencing of cancer-related expression of TCTP.

Another strategy to introduce highly lipophilic moieties into therapeutic ON is their attachment as *O*-2′,3′-ketal nucleolipids at the 5′-end (Figure 9) [451]. Barthélémy et al. connected such a di-C_15_ ketal nucleolipid motif to the 5′-end of a 20-mer PS ASO targeting translationally controlled tumor protein (TCTP) in prostate cancer cells. The mRNA encoding the cancer biomarker TCTP represents a potential therapeutic target [452] as it is overexpressed in several types of cancer including breast [453], lung [454], and particularly castration-resistant prostate cancer (CRPC) [455]. TCTP has been shown to be involved in cell cycle progression [456] and inhibition of programmed cell death [457]. In the advanced stages of prostate cancer, emerging resistance against adjuvant hormone therapy with androgens requires surgery or chemotherapy [458,459] with poor prognosis. An efficient cellular targeting and delivery of a suitable therapeutic ON might be an approach to overcome these issues. This can be achieved by attaching 2′,3′-*O*-(16-hentriacontanylidene)-uridine to the ON in an unusual head-to-head fashion (5′-5′) via its 5′-phosphoramidite. This remarkable structure tends to promote self-aggregation into micelles, thus improving delivery without additional transfecting agents via micropinocytosis in the same manner (98%) as oligofectamine polyplexes of an unmodified ASO (100%) in PC3 cells, but with reduced toxicity (relative to the non-conjugated PS ASO) in nude mice. Thus, the expression of TCTP was downregulated by 90% at 70 nM with the nucleolipid-modified PS ASO, representing a 2-fold increase in silencing activity relative to the nucleolipid-free reference ASO.

## 5. Conclusions

In summary, the conjugation of small molecules to oligonucleotides represents an exciting and promising approach to improve the pharmacokinetic and therefore the therapeutic limitations of ON-derived drug candidates. The attachment of small molecules to ON can furnish improved cellular uptake of the resultant conjugates as well as their enhanced endosomal release. The former can be achieved by exploitation of specific receptor interactions or by conjugation of the ON with lipophilic chemical entities. The latter represents an opportunity to overcome one of the main hurdles in the development of potent therapeutic ON, i.e., an efficient escape from the endosomal machinery into the cytosol. In comparison to drug formulations with transfecting agents, the conjugation of ON with small molecule-derived targeting units has the potential to afford ‘self-delivery’ of the conjugates, thus avoiding the use of potentially toxic transfecting agents. Hence, the development of ON-small molecule conjugates should be considered a therapeutic principle with a promising future, and further innovations in this field can be anticipated.
